# Comparative transcriptomics reveals that a novel form of phenotypic plasticity evolved via lineage‐specific changes in gene expression

**DOI:** 10.1002/ece3.10646

**Published:** 2023-10-20

**Authors:** Andrew J. Isdaner, Nicholas A. Levis, David W. Pfennig

**Affiliations:** ^1^ Department of Biology University of North Carolina Chapel Hill North Carolina USA; ^2^ Department of Biology Indiana University Bloomington Indiana USA

**Keywords:** developmental plasticity, gene expression, novelty, phenotypic plasticity, spadefoot, transcriptomics

## Abstract

Novel forms of phenotypic plasticity may evolve by lineage‐specific changes or by co‐opting mechanisms from more general forms of plasticity. Here, we evaluated whether a novel resource polyphenism in New World spadefoot toads (genus *Spea*) evolved by co‐opting mechanisms from an ancestral form of plasticity common in anurans—accelerating larval development rate in response to pond drying. We compared overlap in differentially expressed genes between alternative trophic morphs constituting the polyphenism in *Spea* versus those found between tadpoles of Old World spadefoot toads (genus *Pelobates*) when experiencing different pond‐drying regimes. Specifically, we (1) generated a de novo transcriptome and conducted differential gene expression analysis in *Spea multiplicata*, (2) utilized existing gene expression data and a recently published transcriptome for *Pelobates cultripes* when exposed to different drying regimes, and (3) identified unique and overlapping differentially expressed transcripts. We found thousands of differentially expressed genes between *S*. *multiplicata* morphs that were involved in major developmental reorganization, but the vast majority of these were not differentially expressed in *P*. *cultripes*. Thus, *S*. *multiplicata*'s novel polyphenism appears to have arisen primarily through lineage‐specific changes in gene expression and not by co‐opting existing patterns of gene expression involved in pond‐drying plasticity. Therefore, although ancestral stress responses might jump‐start evolutionary innovation, substantial lineage‐specific modification might be needed to refine these responses into more complex forms of plasticity.

## Graphical abstract

We evaluated whether a novel resource polyphenism in New World spadefoot toads evolved by co‐opting mechanisms from an ancestral form of plasticity (accelerating larval development rate in response to pond drying) common in anurans using differential gene expression analysis. The vast majority of differentially expressed genes in the New World spadefoot toads were not differentially expressed in the Old World spadefoot toad species. The novel polyphenism thus seems to have evolved primarily through lineage‐specific changes in gene expression rather than via co‐option.

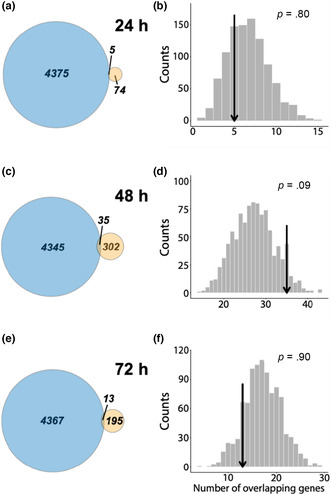

## 1 INTRODUCTION

Phenotypic plasticity is an intrinsic property of life (Nijhout, 2003; Pfennig, 2021a; Sultan, 2021). Indeed, all major groups of organisms—from bacteria to mammals—can respond to environmental variation by undergoing reversible or irreversible shifts in some aspects of their phenotype, including (at the molecular level) gene expression (reviewed in Sultan, 2021). Moreover, plasticity is critical to ecology and evolution (Pfennig, 2021b), having been implicated in mediating species interactions and coexistence (Agrawal, 2001; Hendry, 2016; Hess et al., 2022; Pfennig & Pfennig, 2012; Turcotte & Levine, 2016); evolutionary innovation (Levis & Pfennig, 2021; Moczek et al., 2011); speciation and adaptive radiation (Pfennig et al., 2010; Schneider & Meyer, 2017; Susoy et al., 2016; West‐Eberhard, 1989, 2003; Wund et al., 2008); and macroevolutionary transitions in individuality (Davison & Michod, 2021).

Among the most spectacular forms of plasticity are polyphenisms (sensu Michener, 1961), the occurrence of multiple, discrete environmentally induced phenotypes in a single population. The evolution of a polyphenism has long been viewed as a critical phase in major, lineage‐specific innovations (Levis & Pfennig, 2021; Mayr, 1963; Moczek et al., 2011; Nijhout, 2003; West‐Eberhard, 1989, 2003). Polyphenism promotes innovation by facilitating the accumulation of cryptic genetic variation (Falconer & Mackay, 1996; Gianola, 1982; Reid & Acker, 2022; Roff, 1996). Cryptic genetic variation, in turn, fuels plasticity‐led evolution, which occurs when selection promotes evolutionary change by acting on quantitative genetic variation exposed to selection by environmental changes and plasticity (reviewed in Levis & Pfennig, 2021).

In contrast to these well‐characterized evolutionary consequences of polyphenism, the origins of polyphenism need to be better understood. Generally, polyphenisms are thought to arise when disruptive selection acts on continuously varying plasticity (a reaction norm) and molds it into discrete phenotypes (Pfennig, 2021a). However, the developmental and genetic processes that promote the evolutionary refinement of ancestral plasticity into an adaptive polyphenism require greater explanation (Levis & Ragsdale, 2022; Sommer, 2020). A leading hypothesis is that a novel polyphenism evolves by redeploying existing developmental machinery (Abouheif & Wray, 2002; Bhardwaj et al., 2020; Hanna & Abouheif, 2022; Projecto‐Garcia et al., 2017; Sommer, 2020; Suzuki & Nijhout, 2006). For example, the genetic and developmental underpinnings of a resource polyphenism in the nematode, *Pristionchus pacificus*, partially overlap with both the mechanisms controlling an ancestral form of facultative diapause in which larvae develop into an environmentally resistant “dauer” form (Bento et al., 2010; Casasa et al., 2021; Ogawa et al., 2009) and with conserved starvation‐response genes (Casasa et al., 2021). Thus, the evolution of a polyphenism might co‐opt mechanisms underlying ancestral plastic responses to stressful environmental conditions (for a review of stress‐induced co‐option driving novelty, see Love & Wagner, 2022).

Alongside such shared mechanisms, unique (i.e., lineage‐specific) evolutionary change also contributes to novel forms (Babonis et al., 2016; Cabrales‐Orona & Délano‐Frier, 2021; Jasper et al., 2015; Johnson, 2018; Khalturin et al., 2009). For example, a novel locomotory trait in water striders, *Rhagovelia* spp., that permitted them to fill an unoccupied niche involved lineage‐specific molecular evolution (Santos et al., 2017). Similarly, the resource polyphenism in diplogastrid nematodes mentioned above involves lineage‐specific evolutionary changes in key regulatory genes (Biddle & Ragsdale, 2020; Ragsdale et al., 2013).

Co‐option and non‐shared, lineage‐specific evolution most likely work together to shape the evolution of complex phenotypes, including those associated with polyphenisms. However, more work is needed to understand better the extent to which co‐option versus lineage‐specific changes underlie the evolution of novel plasticity. Moreover, given that plasticity may also facilitate the origins of novel complex traits (see above), such studies promise to provide important insights into the factors that promote evolutionary innovation. A first step in answering this question is to identify patterns of gene expression that are unique to a derived form of plasticity and not shared with more general forms of plasticity. Such lineage‐specific expression patterns could suggest either the broad elaboration of existing forms of plasticity or the evolution of novel forms of plasticity. Future investigations would then be needed to distinguish between these two possibilities.

To begin to address this need, we sought to characterize the extent to which a derived resource polyphenism is mediated at the molecular level by lineage‐specific changes versus mechanisms shared with ancestral plastic responses. To do so, we evaluated whether derived and ancestral forms of plasticity overlap in gene expression patterns. We focused on gene expression for three reasons. First, nearly all forms of plasticity are underlain by differences in gene expression (Goldstein & Ehrenreich, 2021; Renn & Schumer, 2013). Second, gene expression data provide abundant information (Price et al., 2022), which can offer additional insights into underlying mechanisms. Finally, the growing body of transcriptomic data enables comparative approaches needed to examine lineage‐specific versus co‐opted evolution. Indeed, as described below, a key feature of our study utilized existing gene expression data.

**FIGURE 1:**
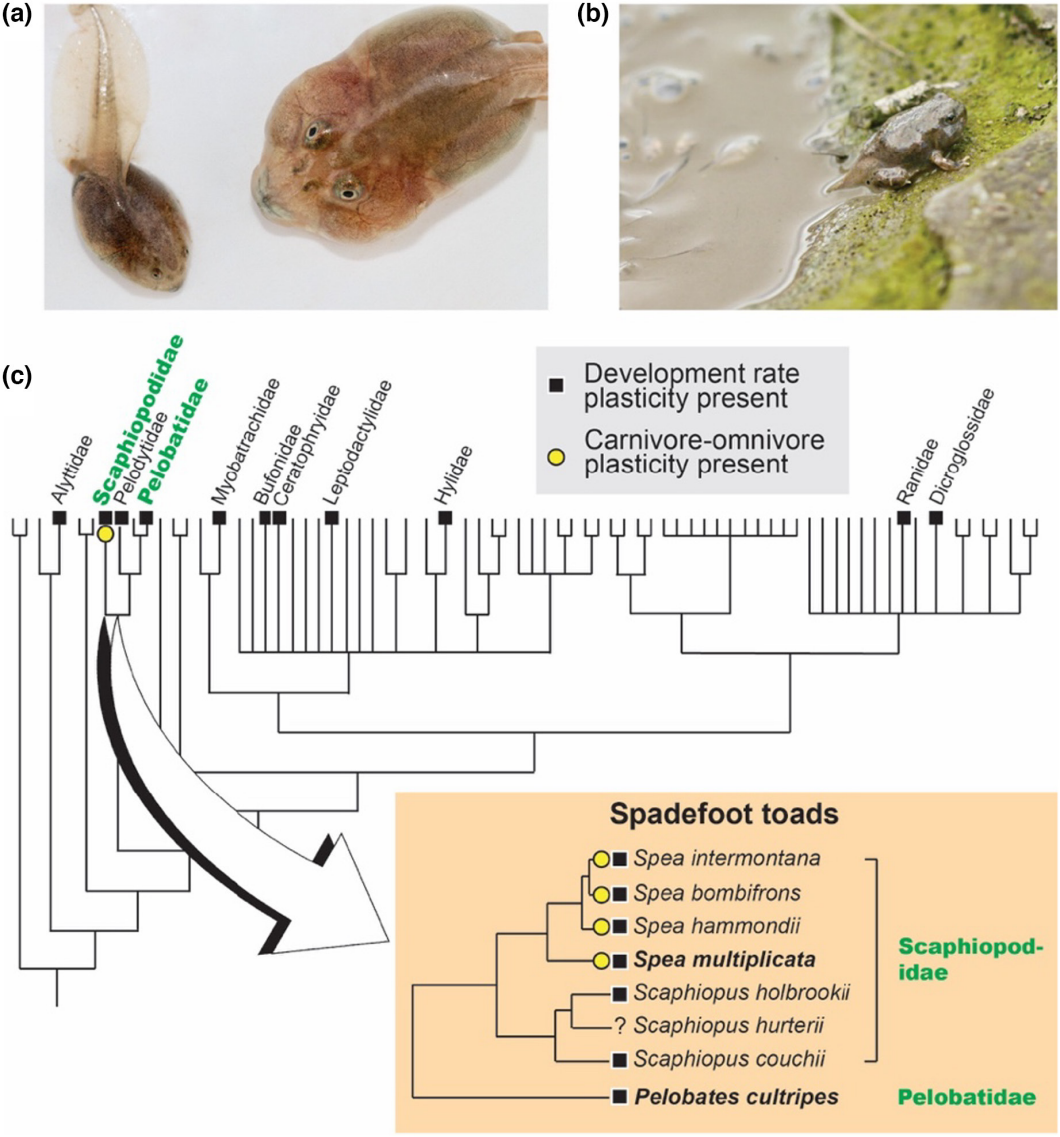
Two forms of plasticity in anuran tadpoles. (a) *Spea* tadpoles (like these *S*. *multiplicata*) have evolved a resource polyphenism, in which they develop into either an omnivore morph (*left*) or, if they are exposed to live prey, a distinctive carnivore morph (*right*). (b) Tadpoles of many anuran species can also facultatively accelerate development in response to pond drying. Here, a *S*. *multiplicata* metamorph escapes a drying pond. (c) Although carnivore‐omnivore plasticity occurs only in *Spea* (family Scaphiopodidae; *open circle*), development‐rate plasticity has been reported in at least 11 anuran families (*filled squares*), suggesting it may have preceded carnivore‐omnivore plasticity (names are shown only for anuran families in which either form of plasticity has been reported). This study focuses on *Spea multiplicata* (Scaphiopodidae) and *Pelobates cultripes* (Pelobatidae; bold font). Phylogeny of anuran families from AmphibiaWeb (2016); Phylogeny of spadefoot toads from Zeng et al. (2014; note: the phylogeny shown here shows only one species of Old World spadefoots in the family Pelobatidae); phylogenetic distribution of carnivore‐omnivore plasticity from Ledón‐Rettig et al. (2008); phylogenetic distribution of development‐rate plasticity from Richter‐Boix et al. (2011), with additional records from Fan et al. (2014), Székely et al. (2017), and Venturelli et al. (2022).

Our focal species, the Mexican spadefoot toad, *Spea multiplicata*, has evolved a novel form of plasticity: a larval resource polyphenism (Ledón‐Rettig & Pfennig, 2011; Pfennig, 1992a; Figure 1a). *Spea* tadpoles typically develop into an “omnivore” morph, which eats detritus, algae, and plankton. However, if they are exposed to live prey early in life (such as fairy shrimp or other tadpoles; Harmon et al., 2023; Levis et al., 2015; Pfennig, 1990), some individuals express an alternative “carnivore” morph (Figure 1a). This novel phenotype—which has evolved only in the genus *Spea* (Ledón‐Rettig et al., 2008)—develops faster than the omnivore morph (de la Serna Buzon et al., 2020; Pfennig, 1992a) and appears to be the analog to developmentally accelerated forms found in other anurans (Pfennig, 1992b). Moreover, the carnivore morph is thought to have arisen when pre‐existing (ancestral within Scaphopodidae) trophic plasticity was refined by selection into an adaptive phenotype as part of a polyphenism (reviewed in Levis & Pfennig, 2019). Recent studies have found that this polyphenism entails changes to lipid metabolism, cholesterol and steroid biosynthesis, and peroxisome form and function (Levis et al., 2020, 2021, 2022). Interestingly, many of these same processes mediate another, much more common form of plasticity in anurans: the ability to facultatively accelerate development in response to pond drying (Figure 1b).

In a shrinking pond, the tadpoles of many anuran species can facultatively initiate metamorphosis and thereby escape the stressful conditions of higher competition and desiccation. Such developmental acceleration occurs throughout the anuran phylogeny (Figure 1c), suggesting it is an ancestral form of plasticity. Of relevance to our study, another research team recently investigated the transcriptomic bases of this plasticity in *Pelobates cultripes*, a European spadefoot that is among the closest relatives of *Spea* (Figure 1c). Notably, this team found that this plasticity involves changes to lipid metabolism, cholesterol, and steroid biosynthesis (Liedtke et al., 2021)—all of which were implicated in mediating *Spea*'s resource polyphenism (Levis et al., 2020, 2021, 2022).

Based on this overlap in mechanisms between the two forms of plasticity, and the fact that the carnivore morph develops faster than the omnivore morph, we hypothesized that being able to accelerate development (an ancestral form of plasticity) contributed, at least in part, to the evolution of *Spea*'s resource polyphenism (a derived form of plasticity; Figure 1c). If this resource polyphenism did indeed evolve using shared mechanisms from the more ancestral pond‐drying plasticity, we predicted that we would find significant overlap in differentially expressed genes between these two forms of plasticity. To test this prediction, we used comparative transcriptomics to determine the extent to which gene expression differences between *S*. *multiplicata* carnivores and omnivores overlap with gene expression responses to pond drying in *P*. *cultripes*. We did so by making use of *S*. *multiplicata* carnivores and omnivores generated for a previously published transcriptomic study (Levis et al., 2021) and recently published transcriptomic data from *P*. *cultripes* (Liedtke et al., 2021). In this way, we leveraged existing data to evaluate whether a novel form of phenotypic plasticity evolved by lineage‐specific changes or by co‐opting mechanisms from ancestral forms of plasticity.

## 2 METHODS

### 2.1 Acquisition of experimental tadpoles

*Spea multiplicata* carnivores and omnivores were generated for a previously published transcriptomic study (Levis et al., 2021). For that study, three pairs of Mexican spadefoot toads (*S. multiplicata*) were collected in amplexus from a newly formed, temporary pond near Portal Arizona (“po2‐N Pond”) and transported to the nearby Southwestern Research Station to breed. For each sibship, tadpoles were divided into five boxes of 80 tadpoles each and fed fish food (10 mg daily to mimic pond detritus; Pfennig et al., 1991) as well as live fairy shrimp and live *Scaphiopus couchii* tadpoles. Competition, shrimp consumption, and tadpole consumption contribute to the development of carnivores (Levis et al., 2015, 2017; Pfennig, 1990, 1992b), but not all individuals that experience these cues develop into a carnivore; some remain omnivores even after experiencing carnivore‐inducing conditions (Pfennig, 1990). When the tadpoles were 10d old, five omnivores and five carnivores per sibship were randomly sampled, euthanized with a 0.8% aqueous tricaine methanesulfonate (MS‐222) solution, and placed in a microcentrifuge tube filled with RNAlater. These samples remained at room temperature for 24 h to allow RNAlater penetration and then were frozen at −20°C until being shipped to the University of North Carolina overnight on dry ice. Samples were held at −80°C until use in the present study.

### 2.2 RNA extraction, library preparation, and sequencing

We extracted whole‐body total RNA from three carnivore tadpoles and three omnivore tadpoles from each of three sibships, for a total of nine carnivores and nine omnivores. We used whole‐tadpole samples to match the approach used in Liedtke et al. (2021) for *P*. *cultripes* as closely as possible. Total RNA was extracted using the TRIzol Plus RNA Purification Kit (Invitrogen, #12183555), followed by treatment with DNase. We determined RNA purity for each sample using a NanoDrop 2000 (Thermo Scientific) and quantified total RNA on a Qubit 4 using the RNA HS Assay Kit (Thermo Scientific). The RNA samples were shipped to Novogene, where sample QC, library preparation, and sequencing were performed. We generated 150‐PE reads using a NovaSeq 6000 sequencer (Illumina). Of the initial 18 samples, 14 passed quality control and were sequenced. All four samples that were not sequenced were carnivores, with three from a single family. That family was included in generating the de novo transcriptome but excluded from all differential expression analyses (see below).

### 2.3 Generation of de novo transcriptome and quality assessment

The sequence data was examined for quality using “FastQC” (Andrews, 2010). After combining all reads, we utilized “Trinity” v2.8.6 (Haas et al., 2013) to trim reads, perform in silico normalization, and then generate a draft assembly of the *S*. *multiplicata* tadpole whole‐body transcriptome (“Trinity” flags used: ‐‐trimmomatic ‐‐normalize_max_read_cov 50). Trimming was performed within the “Trinity” call using default Trimmomatic settings: SLIDINGWINDOW:4:5 LEADING:5 TRAILING:5 MINLEN:25 (Bolger et al., 2014).

We examined the quality of the transcriptome for both read representation and completeness of gene content. To investigate read representation, we mapped normalized read pairs back onto the transcriptome using Bowtie2 v2.4.5 (Langmead & Salzberg, 2012) to determine the percentage of all paired reads represented in the transcriptome assembly. To examine gene content completeness, we first used “BUSCO” v.5.2.2 (Manni et al., 2021) with the “tetrapoda‐odb10 database” as our reference, which allows us to examine whether highly conserved tetrapod genes are present in the assembly. We also ran “blastx” against both the SwissProt database and the *Xenopus tropicalis* proteome, using an Evalue threshold of ≤1e^−20^, to identify sequences that highly match other related transcriptomes.

### 2.4 Functional annotation of transcriptome

Functional annotation of the transcriptome assembly was performed using “Trinotate” v.3.2.1 (Bryant et al., 2017). “Trinotate” combines various annotations into a single output; each annotation is performed individually. We first identified transcript sequences with similarities to known proteins using “blastx” (Evalue cutoff ≤1e^−5^) against the SwissProt database and a subset of the SwissProt database consisting of only human genes. We next sought likely coding regions using “TransDecoder” (https://github.com/TransDecoder). The resulting putative coding regions were queried against the complete SwissProt database and a subset of the SwissProt database consisting of only vertebrates using “blastp” (Evalue cutoff ≤1e^−5^). We additionally searched for conserved protein domains using “HMMER” (http://hmmer.org) against the Pfam database (Finn et al., 2015). We used “SignalP” v4.1 (Petersen et al., 2011) to predict signal peptides and “TmHMM” v2.0 (https://services.healthtech.dtu.dk/service.php?TMHMM‐2.0) to predict transmembrane regions. As a final step, we applied gene ontology (GO) terms, as well as KEGG (Kyoto Encyclopedia of Genes and Genomes; http://www.genome.jp/kegg/) and EggNOG (Huerta‐Cepas et al., 2015) annotations as provided by “Trinotate,” to each transcript in the assembly. The resulting annotation database produced by “Trinotate” was examined using the R package “TrinotateR” (https://github.com/cstubben/trinotateR).

We next estimated transcript abundance using “kallisto” v0.46.1 (Bray et al., 2016) and subsequently excluded all transcripts with less than one transcript per million (<1 TPM) from the transcriptome assembly since transcripts with very low expression levels are of dubious biological relevance. The assembly was next evaluated using NCBI's VecScreen to filter out possible vector, adapter, and/or primer contamination of the transcriptome. We additionally used the “MCSC” decontamination method (Lafond‐Lapalme et al., 2016) with the target phylum Chordata to attempt to filter out any inferred non‐chordate transcripts. Further, the resulting transcripts were compared to the “nt” database using “blastx” (Evalue cutoff ≤1e^−5^), and all transcripts with best matches outside Chordata were removed. Transcripts that had no match were retained. Finally, the assembly was blasted against the NCBI UniVec database using standard VecScreen parameters, filtering out transcripts with a match below an Evalue threshold of 1e^−7^. Thus, we applied extensive quality control filters to produce our final transcriptome.

### 2.5 Analysis of differential gene expression

For our differential expression analyses, we only included *S*. *multiplicata* samples from families with omnivore and carnivore sequencing data (i.e., families 5 and 11). We first estimated transcript abundance at the Trinity “gene” level using “kallisto” v0.46.1 (Bray et al., 2016). Utilizing “edgeR” v3.38.1 (McCarthy et al., 2012; Robinson et al., 2010), we examined the clustering of individuals by morph using multi‐dimensional scaling of log2 counts per million. “edgeR” was then used to identify differentially expressed genes (DEGs) between carnivores and omnivores, with family as a covariate. We considered all genes with a false discovery rate of *q* < 0.05 significantly differentially expressed. This set of differentially expressed genes likely constitutes a set of downstream “effectors” that maintain or allow functioning of the alternative phenotypes, as opposed to upstream master regulatory genes.

We implemented the same procedure to identify differentially expressed genes in response to pond drying in *P*. *cultripes*, which undergoes plastic developmental acceleration in response to drying pond conditions. The raw sequence data for *P*. *cultripes* was accessed from the NCBI Sequence Read Archive (SRA; SRP161446) and the transcriptome from the NCBI Transcriptome Shotgun Assembly database (TSA; GHBH01000000) under BioProject PRJNA490256. Previous analysis of this data (Liedtke et al., 2021) identified differentially expressed genes between a high‐water control and three different time points in a low‐water treatment. We re‐analyzed differential gene expression for each pair of high‐water control and low‐water treatment time points individually. Doing so allowed us to evaluate how each timepoint corresponds (in terms of shared differentially expressed genes) to differential expression between carnivores and omnivores while analyzing each data set identically.

### 2.6 Functional annotation of differentially expressed genes

We examined each species' differentially expressed genes for functional enrichment using g:Profiler in its web‐based implementation (Raudvere et al., 2019). We conducted this analysis using annotations for the human proteome as the background domain. For *P*. *cultripes*, this analysis was performed for differentially expressed genes in each pairwise set of high‐water control and low‐water treatment time points. We corrected for multiple testing using g:Profiler's g:SCS algorithm. We examined ontologies and pathways from the GO:Biological Process, KEGG, and Reactome databases.

### 2.7 Analysis of overlap in differentially expressed genes and functional annotations between *Spea* and *Pelobates*

Because sequence differences across the two species might lead to similar sequences matching different annotations, we performed a reciprocal best‐hit annotation using “blastn” to generate a list of matching sequences between *S*. *multiplicata* and *P*. *cultripes* (as opposed to comparing best‐hit annotations to one another, since the best match may be a different ortholog or in a different reference species across the two spadefoot species). We then performed a second differential expression analysis using “edgeR” for each species using this direct cross‐species annotation.

To address the question of whether *S*. *multiplicata* utilizes an existing plastic response to desert conditions, we queried the list resulting from the differentially expressed gene analysis in *S*. *multiplicata* against the corresponding list from each pairwise comparison in *P*. *cultripes* (i.e., between each low‐water timepoint and the high‐water control) to determine the number of genes overlapping between the two species contrasts. We performed permutation tests at each time point to evaluate if the number of overlapping DEGs was greater than expected by random chance. To conduct these tests, we randomly sampled genes from the expression‐filtered transcriptome of each species corresponding to: (1) the number of genes differentially expressed in *S*. *multiplicata*, and (2) the number of genes differentially expressed in *P*. *cultripes*. We then examined the number of overlapping genes from each permutation on a pairwise basis corresponding to the original analyses and determined the number of permutations that equaled or exceeded the equivalent value from the actual data to calculate a measure of statistical significance.

We next identified the differentially expressed genes in *S*. *multiplicata* that were not significantly differentially expressed at each timepoint in *P*. *cultripes* or that did not align to genes in *P*. *cultripes*. These analyses examine whether (1) constitutively expressed genes in *P*. *cultripes* have acquired new differential expression patterns in *S*. *multiplicata* as a component of its polyphenism, and (2) the *S*. *multiplicata* polyphenism possesses unique genes not found in *P*. *cultripes*, respectively. Finding genes in either of these two classes would be consistent with lineage‐specific evolution of gene expression in *S*. *multiplicata*.

Finally, we analyzed overlapping functional annotation using g:Profiler on the overlapping gene sets in each time period and comparison (DEGs vs. DEGs or DEGs vs. non‐significant genes) and for the set of genes unique to *S*. *multiplicata*'s polyphenism using annotations from the GO: Biological Process, KEGG, and Reactome datasets.

## 3 RESULTS

### 3.1 Transcriptomics of *Spea* morphs

Conducting comparative gene expression required the de novo assembly of a transcriptome for *S*. *multiplicata* tadpoles. To do so, we generated between 16.4 and 24.5 million 150‐PE reads (mean of 20.8 million reads), for a total of 582.0 million 150‐PE reads, across the 14 sequenced samples. After in silico normalization, 20.4 million pair‐end reads (3.5% of the total reads) served as input for assembling the *S*. *multiplicata* transcriptome. The output from “Trinity” consisted of 457,153 transcript contigs (median length = 430 bp), which clustered into 310,955 “genes” (that is, clusters of transcripts with shared sequence content). We mapped 97.3% of all paired reads onto the transcriptome de novo assembly (Table 1). BUSCO analysis indicates near‐complete sequence information for 93.1% of the genes in the “tetrapoda_odb10” database, with just 2.0% of genes fragmented and 4.9% missing (Table 2). Aligning the *S*. *multiplicata* transcriptome to the SwissProt database using “blastx” resulted in 15,004 SwissProt proteins represented by nearly full‐length transcripts (>80% alignment coverage), and a similar analysis comparing to the *X*. *tropicalis* proteome revealed 15,882 proteins represented by nearly full‐length transcripts, out of the 22,282 genes and 37,716 proteins found in the *X*. *tropicalis* proteome. These values compare favorably to the number of nearly full‐length transcripts aligned to each protein database in the recently assembled *P*. *cultripes* transcriptome (13,645 and 12,715 proteins, respectively; Liedtke et al., 2019). These results indicate that we have generated a high‐quality transcriptome for whole‐tadpole *S*. *multiplicata*, at least as complete as those previously assembled for other species of spadefoot toads (Liedtke et al., 2019).

Multiple functional annotations of the *S*. *multiplicata* transcriptome served as input for Trinotate (complete annotation in Data S1). A comparison of the transcriptome assembly to the SwissProt database using “blastx” provided a best‐match annotation for 216,650 transcripts (Table 3). When these annotations were subjected to GO analysis, we matched 21,251 unique GO terms (out of 2,042,040 total terms). Prediction of coding regions (CDS) with “TransDecoder” identified 159,127 CDS, representing 51.2% of the Trinity “genes” in the assembly. Comparison of the TransDecoder results against the SwissProt database using “blastp” annotated 115,297 CDS, and a second comparison to the vertebrate‐only subset of SwissProt annotated 113,254 CDS. Other annotations of TransDecoder‐predicted CDS included 99,382 hits against the Pfam database, 11,935 signalP‐predicted peptides, 27,573 TmHMM‐predicted transmembrane proteins, and 152,816 KEGG terms (Table 3). Among sequences that were annotated with vertebrate genes, 26,281 unique proteins from the vertebrate‐only subset of SwissProt were recovered in *S*. *multiplicata*. Parallel analysis of the *P*. *cultripes* transcriptome identified 25,029 unique vertebrate proteins in that species' transcriptome. Between the two species, there were 32,853 unique proteins recovered, with 18,457 (56.2% of the total) shared between the two species, 7824 (23.8%) unique to *S*. *multiplicata*, and 6572 (20.0%) unique to *P*. *cultripes*. After filtering to remove transcripts with low expression (<1 TPM), 70.8% of the transcripts in the transcriptome were retained. VecScreen filtering further reduced the size of the *S*. *multiplicata* transcriptome by 95 transcripts. After conducting “MCSC” decontamination to remove inferred non‐chordate transcripts and manual filtration using the UniVec database, the transcriptome consisted of 288,112 transcripts. In summary, our densely annotated, filtered transcriptome allows for robust and informative downstream analyses of gene expression patterns and other transcriptomic inquiries.

**TABLE 1:** Transcriptome assembly statistics for *Spea multiplicata*.

*S*. *multiplicata* transcriptome assembly	
Total raw reads	582,031,892
In silico normalized reads	20,401,976
Trinity transcripts in assembly	547,153
Trinity “genes” in assembly	310,955
Read pairs aligned to the assembly	97.3%
Proper pair reads aligned to the assembly	92.1%
N50 of transcripts	2676 bp
N50 of longest isoform per “gene”	855 bp
Size of total transcriptome	500,433,975 bp
Size of transcriptome only incl. longest isoform per “gene”	195,537,278 bp
Median size of transcripts	430 bp
Median size of longest isoform per “gene”	342 bp
Average size of transcripts	1094.7 bp
Average size of longest isoform per “gene”	628.8 bp

*Note*: Trinity outputs are provided at both the transcript and “gene” levels.

**TABLE 2:** Gene content completeness assessment of the *Spea multiplicata* transcriptome assembly.

*S*. *multiplicata* transcriptome gene content	
Proteins represented by nearly full‐length transcripts^a^ compared to	
SwissProt	15,004
*Xenopus tropicalis* proteome	15,822
BUSCO results	
Complete	93.1%
Fragmented	2.0%
Missing	4.9%

*Note*: BUSCO was performed using the “tetrapoda‐odb10” database.

^a^ >80% alignment coverage; based on grouped high scoring segment pairs (HSPs) to account for multiple fragments per transcript aligning to a single sequence.

**TABLE 3:** Summary of Trinotate results indicating the number of annotations for unique//total TransDecoder‐predicted candidate genes identified with various tools and databases.

Annotation summary of the *Spea multiplicata* transcriptome assembly	
TransDecoder‐predicted coding regions (ORFs)	159,127
SwissProt protein hits (blastp)	76,407//115,297
SwissProt vertebrates only protein hits (blastp)	74,536//113,254
Pfam hits (HMMER)	64,573//99,382
Predicted peptides (signalP)	3819//11,935
Predicted transmembrane proteins (tmHMM)	16,875//27,573
GO Pfam	2628//61,836
KEGG	38,663//152,816
Transcripts annotated against SwissProt (blastx)	216,650
blastx GO terms (unique//total)	21,251//2,042,040

**FIGURE 2:**
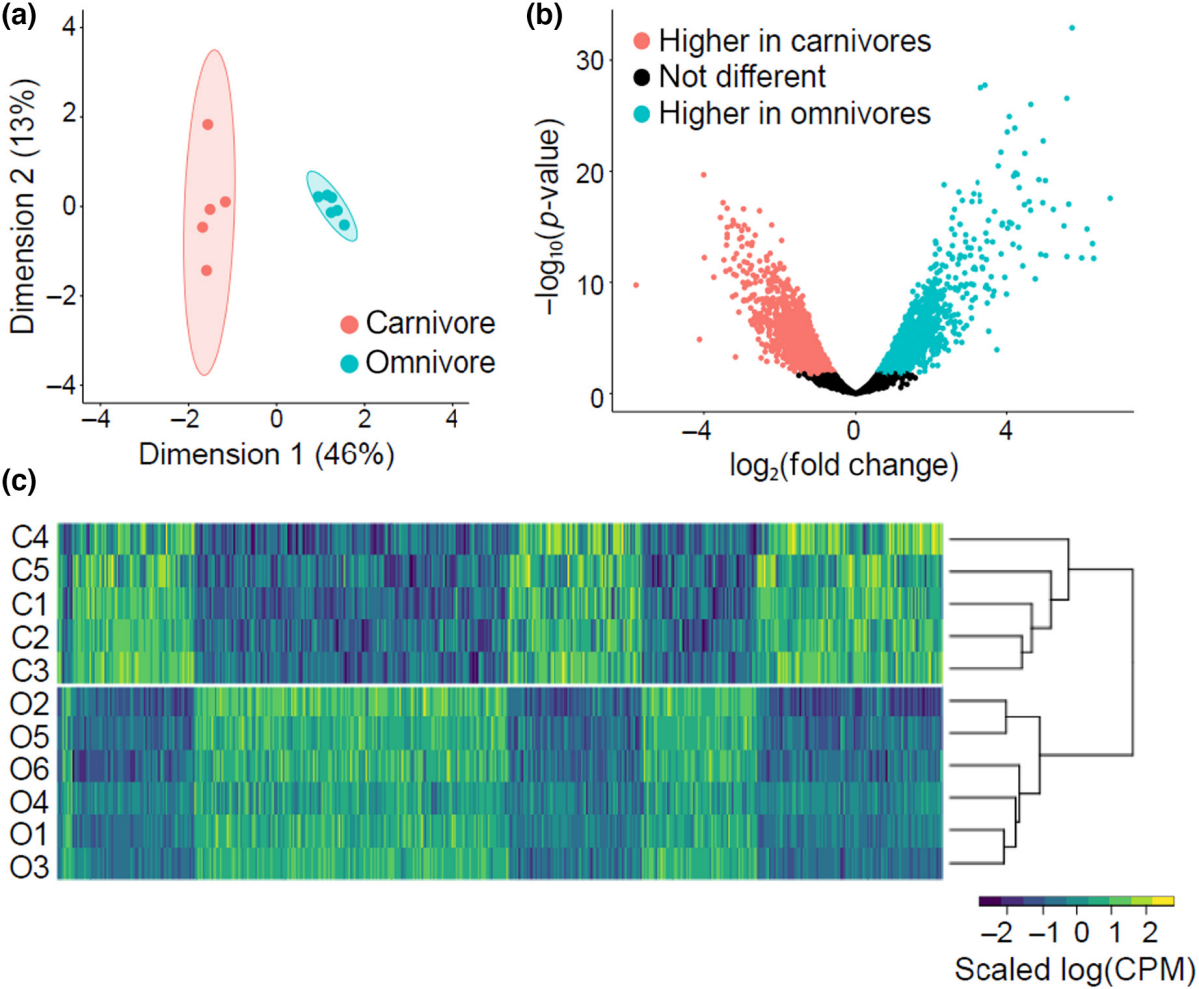
Gene expression patterns within *Spea multiplicata* tadpoles. (a) Multidimensional scaling plots of log_2_‐counts‐per‐million along the first dimensions. (b) Volcano plot of RNA‐seq data at the Trinity “gene” level, where differentially expressed genes with *q* < 0.05 are statistically significant. (c) Heat map of log_2_ counts‐per‐millions for transcripts that show statistically‐significant differential expression between carnivores and omnivores. Carnivore samples are labeled with C1 through C5, omnivore samples are labeled from O1 through O6.

With our transcriptome assembled, we next analyzed gene expression patterns between carnivores and omnivores in *S*. *multiplicata*. Multidimensional scaling analysis on standardized count data (log_2_ CPM) for *S*. *multiplicata* revealed distinct clusters for carnivores and omnivores along the first dimension, accounting for 46% of the variation between the two morphs (Figure 2a). Of the 12,676 genes with expression data across the samples, 2177 had significantly higher expression in omnivores, while 2203 had significantly higher expression in carnivores (FDR < 0.05; Figure 2b, Data S2).

When DEGs are clustered using hierarchical clustering with complete linkage based on expression pattern (Figure 2c), there are six major clusters of DEGs between *S*. *multiplicata* omnivores and carnivores. In total, 34.6% of the total genes were differentially expressed between the morphs. Functional enrichment analysis of these genes resulted in many terms, reflecting many DEGs between morphs (Data S3). Thus, our de novo transcriptome enables the detection of unique gene expression profiles for carnivores and omnivores that differ in functions related to protein metabolism, developmental processes, regulation of cellular processes, cell differentiation, signal transduction, cellular response to chemical stress, and cardiac muscle contraction. The large number of DEGs and wide range of functional categories support the idea that divergence between these two morphs encompasses a complex suite of developmental differences.

### 3.2 Transcriptomics of *P*. *cultripes* developmental acceleration

To compare gene expression between ancestral and derived forms of plasticity, we next performed differential gene expression analysis in *P*. *cultripes* in the same way as in *S*. *multiplicata* (using raw read data previously published in Liedtke et al., 2019). Pairwise differential expression between the 24‐h high water control and each low water treatment (24, 48, or 72 h) in *P*. *cultripes* results in the following (Data S2): (1) in the 24‐h treatment, 79 transcripts were differentially expressed; (2) in the 48‐h treatment, 337 transcripts were differentially expressed; and (3) in the 72‐h treatment, 208 transcripts were differentially expressed. In total, 492 unique transcripts were differentially expressed between the control and all treatments. Functional enrichment analysis of each pairwise comparison (Data S3) revealed: (1) no terms for the 24‐h treatment; (2) cholesterol metabolic processes, lipid metabolic processes, steroid metabolic processes, and steroid biosynthetic processes for the 48‐h treatment; and (3) cholesterol metabolic processes, alcohol metabolic processes, steroid metabolic processes, lipid biosynthetic processes, steroid biosynthetic processes, and regulation of mast cell cytokine production for the 72‐h treatment. Thus, despite the number of DEGs between water level treatments in *P*. *cultripes* being roughly an order of magnitude lower than the number between carnivore and omnivore *S*. *multiplicata*, the DEGs in *P*. *cultripes* are still enriched for particular functional (e.g., gene ontology) categories.

### 3.3 Shared responses between resource polyphenism and developmental acceleration

**FIGURE 3:**
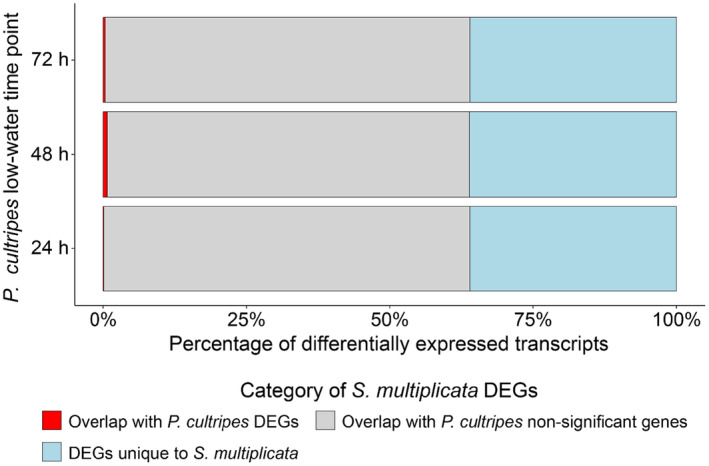
Differentially expressed genes (DEGs) in *Spea multiplicata* in relation to *Pelobates cultripes*. Differentially expressed genes in *S*. *multiplicata* categorized by whether they: (1) overlap with differentially expressed genes in *P*. *cultripes* (red), (2) overlap with genes that are not significantly differentially expressed in *P*. *cultripes* (gray), (3) do not align to any genes in *P*. *cultripes* (light blue).

**FIGURE 4:**
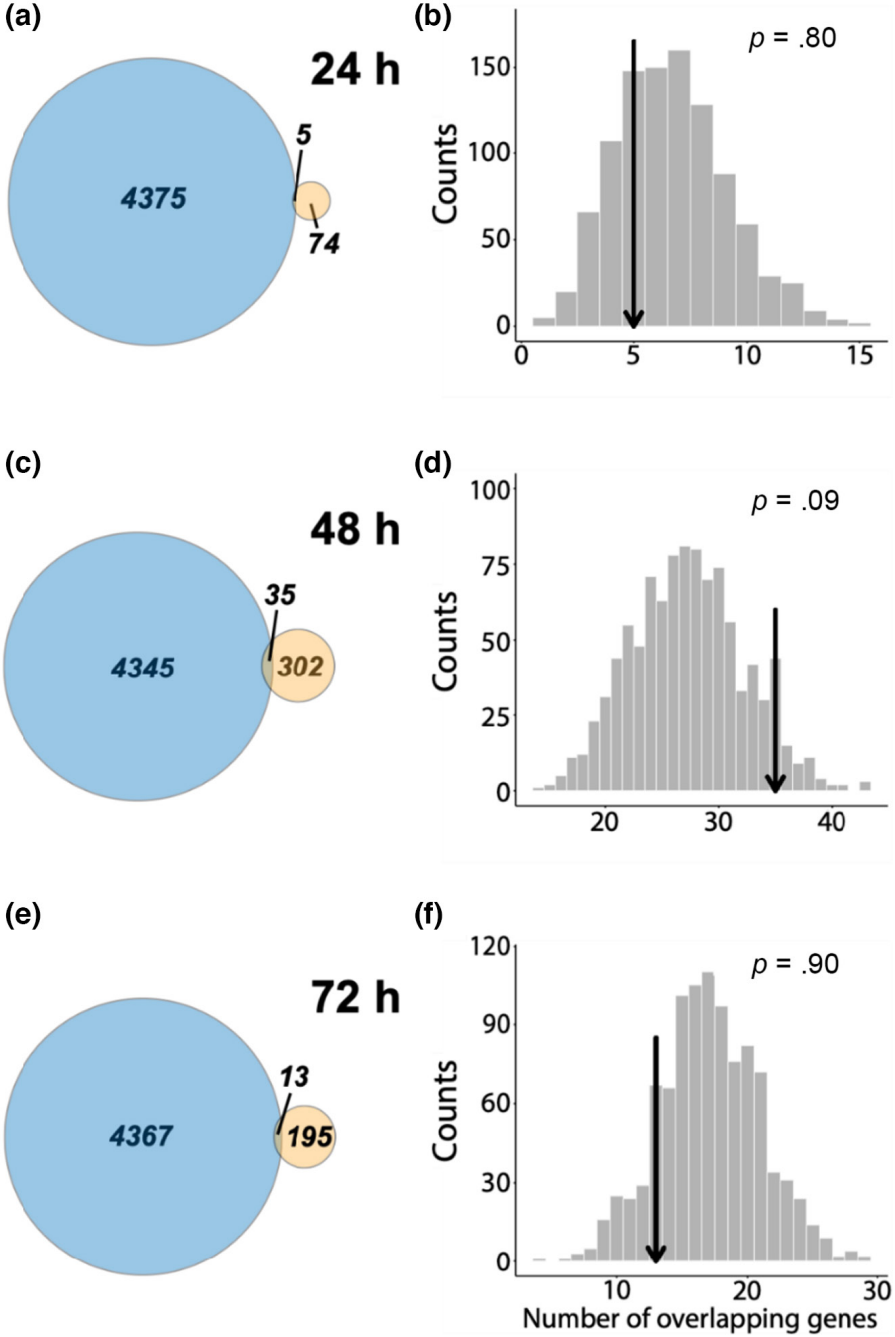
Examining the overlap in differential gene expression between *Spea multiplicata* and *Pelobates cultripes*. Each row depicts a Euler plot of the total number of differentially expressed genes in each species (blue = *S*. *multiplicata*, yellow = *P*. *cultripes*) and a histogram of the number of overlapping genes in 1000 permutations, in each of which the actual number of differentially expressed genes was selected from the expression‐filtered list of genes found in the transcript data from each species, respectively. The observed number of overlaps is marked on each histogram, as is the calculated *p*‐value (the proportion of permutations with more overlapping genes than the observed value). The number of differentially expressed genes between carnivores and omnivores in *S*. *multiplicata* is the same in all three Euler plots. The control for each row is the 24‐h high‐water samples, while the treatments are the samples from 24‐h low water (a, b), 48‐h low water (c, d), and 72‐h low water (e, f).

Comparing the results of the differential gene expression analysis across both species based on the reciprocal‐best‐match annotation, we identified a very limited number of genes that were differentially expressed both between carnivores and omnivores in *S*. *multiplicata* and between high‐water control and low‐water treatments in *P*. *cultripes* (see Table 4; Figure 3). There were five overlapping genes between *S*. *multiplicata* and *P*. *cultripes* when compared to the 24‐h low‐water treatment (Figure 4a), 35 overlapping genes when compared to the 48‐h low‐water treatment (Figure 4c), and 13 overlapping genes when compared to the 72‐h low‐water treatment (Figure 4e). In total, there were 46 unique overlapping genes between those differentially expressed in *S*. *multiplicata* and those in *P*. *cultripes*. Permutation tests indicate that each result is not significantly different from random expectations (24‐h treatment: *p* = .80, 48‐h treatment: *p* = .09; 72‐h treatment: *p* = .90; Figure 3b,d,f), suggesting that there is not a greater than expected number of differentially expressed genes shared between these forms of plasticity.

**TABLE 4:** Differentially expressed genes shared between *Spea multiplicata* (*S*.*m*. below) resource polyphenism and *Pelobates cultripes* (*P*.*c*. below) developmental acceleration (logFC = log_2_(fold‐change), HW = high water, LW = low water, C = carnivore, O = omnivore).

Shared differentially expressed genes								
Human gene ID	Gene name	*P*.*c*. treatment	*P*.*c*. logFC	*P*.*c*. *p*‐value	*S*.*m*. logFC	*S*.*m*. *p*‐value	*P*.*c*. higher expression	*S*.*m*. higher expression
SPSB3	**splA/ryanodine receptor domain and SOCS box protein 3**	**24 h**	**1.41**	**5.77e‐05**	**−1.12**	**3.05e‐04**	**HW**	**C**
HPX	Hemopexin	24 h	−2.02	1.54e‐05	2.60	3.94e‐06	LW	O
PTP4A2	Protein tyrosine phosphatase 4A2	24 h	−1.54	1.56e‐07	−1.19	1.70e‐05	LW	C
ADM2	**Adrenomedullin 2**	**24 h**	**2.04**	**3.23e‐08**	**−2.67**	**4.68e‐08**	**HW**	**C**
TMEM67	Transmembrane protein 67	24 h	−1.35	2.00e‐06	1.45	9.88e‐05	LW	O
CYTL1	Cytokine like 1	48 h	−1.97	4.78e‐06	−2.33	3.80e‐08	LW	C
FGL1	Fibrinogen like 1	48 h	−0.97	2.00e‐04	0.83	1.51e‐02	LW	O
RCN1	Reticulocalbin 1	48 h	1.15	6.08e‐05	−1.39	4.68e‐03	HW	C
MARS1	Methionyl‐tRNA synthetase 1	48 h	1.22	2.35e‐04	−1.00	2.42e‐05	HW	C
ESCO2	Establ. of sister chromatid cohesion N‐acetyltransferase 2	48 h	1.18	3.40e‐04	−1.28	1.95e‐03	HW	C
ARHGEF3	Rho guanine nucleotide exchange factor 3	48 h	−1.36	8.41e‐05	−0.97	3.59e‐05	LW	C
PKLR	**Pyruvate kinase L/R**	**48 h**	**3.80**	**2.40e‐12**	**−0.83**	**9.02e‐03**	**HW**	**C**
FARSA	Phenylalanyl‐tRNA synthetase subunit alpha	48 h	1.47	1.33e‐04	−1.66	2.92e‐09	HW	C
CARS1	Cysteinyl‐tRNA synthetase 1	48 h	1.15	8.07e‐05	−1.29	9.68e‐08	HW	C
SPSB3	**splA/ryanodine receptor domain and SOCS box protein 3**	**48 h**	**2.05**	**3.89e‐05**	**−1.12**	**3.05e‐04**	**HW**	**C**
FCHSD1	FCH and double SH3 domains 1	48 h	−0.82	4.34e‐04	1.40	6.91e‐05	LW	O
PMM1	Phosphomannomutase 1	48 h	0.99	2.25e‐04	1.57	9.36e‐05	HW	O
ADM2	**Adrenomedullin 2**	**48 h**	**1.63**	**1.53e‐04**	**−2.67**	**4.68e‐08**	**HW**	**C**
GP2	Glycoprotein 2	48 h	−1.47	6.76e‐06	1.35	1.09e‐03	LW	O
GART	Phosphoribosylglycinamide formyltransferase	48 h	0.97	3.65e‐04	−1.01	7.71e‐03	HW	C
DHCR24	**24‐dehydrocholestrol reductase**	**48 h**	**3.39**	**4.81e‐09**	**0.91**	**4.60e‐03**	**HW**	**O**
TM7SF2	Transmembrane 7 superfamily member 2	48 h	1.51	1.49e‐04	1.78	1.40e‐07	HW	O
CYP51A1	**Cytochrome P450 family 51 subfamily A member 1**	**48 h**	**3.59**	**3.79e‐15**	**1.97**	**4.01e‐05**	**HW**	**O**
TYR	Tyrosinase	48 h	1.10	1.54e‐05	0.91	3.33e‐03	HW	O
SCD	Stearoyl‐CoA desaturase	48 h	1.96	8.41e‐05	−1.15	5.54e‐03	HW	C
NARS1	Asparaginyl‐tRNA synthetase 1	48 h	1.57	7.01e‐06	−0.93	1.02e‐03	HW	C
MSMO1	**Methylsterol monooxygenase 1**	**48 h**	**3.13**	**3.00e‐09**	**1.95**	**8.59e‐04**	**HW**	**O**
ACSS2	**Acyl‐CoA synthetase short chain family member 2**	**48 h**	**2.14**	**8.51e‐05**	**2.08**	**4.99e‐11**	**HW**	**O**
TP53RK	TP53 regulating kinase	48 h	1.78	4.21e‐07	−0.81	4.36e‐03	HW	C
PHGDH	Phosphoglycerate dehydrogenase	48 h	1.62	2.55e‐07	−2.32	4.66e‐10	HW	C
C9ORF64	Q‐nucleotide N‐glycosylase 1	48 h	2.74	3.11e‐04	1.06	2.74e‐03	HW	O
PSAT1	Phosphoserine aminotransferase 1	48 h	1.92	1.66e‐06	0.93	7.32e‐03	HW	O
CYP4F22	Cytochrome P450 family 4 subfamily F member 22	48 h	2.01	1.22e‐09	0.92	2.41e‐03	HW	O
IPO4	Importin 4	48 h	1.19	3.70e‐04	−1.04	3.51e‐04	HW	C
MAD2L1BP	MAD2L1 binding protein	48 h	1.94	4.32e‐06	−1.55	4.14e‐03	HW	C
ACAT2	Acetyl‐CoA acetyltransferase 2	48 h	1.25	2.45e‐04	0.92	7.02e‐03	HW	O
STOML2	Stomatin like 2	48 h	1.85	1.44e‐05	−1.36	1.67e‐06	HW	C
FEN1	Flap structure‐specific endonuclease 1	48 h	1.20	3.67e‐04	−0.95	6.95e‐03	HW	C
CRYGD	Crystallin gamma D	48 h	1.57	7.35e‐05	0.81	1.12e‐02	HW	O
ADK	Adenosine kinase	48 h	1.46	7.77e‐05	−0.78	1.64e‐02	HW	C
PKLR	**Pyruvate kinase L/R**	**72 h**	**3.00**	**4.50e‐07**	**−0.83**	**9.02e‐03**	**HW**	**C**
TMEM97	Transmembrane protein 97	72 h	1.23	2.22e‐04	1.50	2.96e‐06	HW	O
CALU	Calumenin	72 h	−1.94	1.88e‐04	−0.92	1.68e‐02	LW	C
HSD3B7	Hydroxy‐delta‐5‐steroid dehydrogenase	72 h	1.52	2.76e‐04	1.66	8.26e‐05	HW	O
AOC1	Amine oxidase copper containing 1	72 h	−2.05	8.82e‐06	1.56	3.35e‐05	LW	O
CYP51A1	**Cytochrome P450 family 51 subfamily A member 1**	**72 h**	**3.03**	**5.33e‐10**	**1.97**	**4.01e‐05**	**HW**	**O**
DHCR24	**24‐dehydrocholestrol reductase**	**72 h**	**2.84**	**5.94e‐07**	**0.91**	**4.60e‐03**	**HW**	**O**
MSMO1	**Methylsterol monooxygenase 1**	**72 h**	**2.61**	**4.18e‐07**	**1.95**	**8.59e‐04**	**HW**	**O**
PISD	Phosphatidylserine decarboxylase	72 h	1.72	2.20e‐04	−1.02	9.57e‐05	HW	C
ACSS2	**Acyl‐CoA synthetase short chain family member 2**	**72 h**	**2.29**	**4.74e‐05**	**2.08**	**4.99e‐11**	**HW**	**O**
MARCHF8	Membrane associated ring‐CH‐type finger 8	72 h	4.59	5.64e‐08	1.37	4.23e‐04	HW	O
HMGCR	3‐hydroxy‐3‐methylglutaryl‐CoA reductase	72 h	2.00	1.17e‐04	1.43	1.80e‐05	HW	O
ATL2	Atlastin GTPase 2	72 h	3.45	4.02e‐06	0.62	8.75e‐03	HW	O

*Note*: Genes that appear in more than one *P*.*c*. treatment are labeled in bold.

**FIGURE 5:**
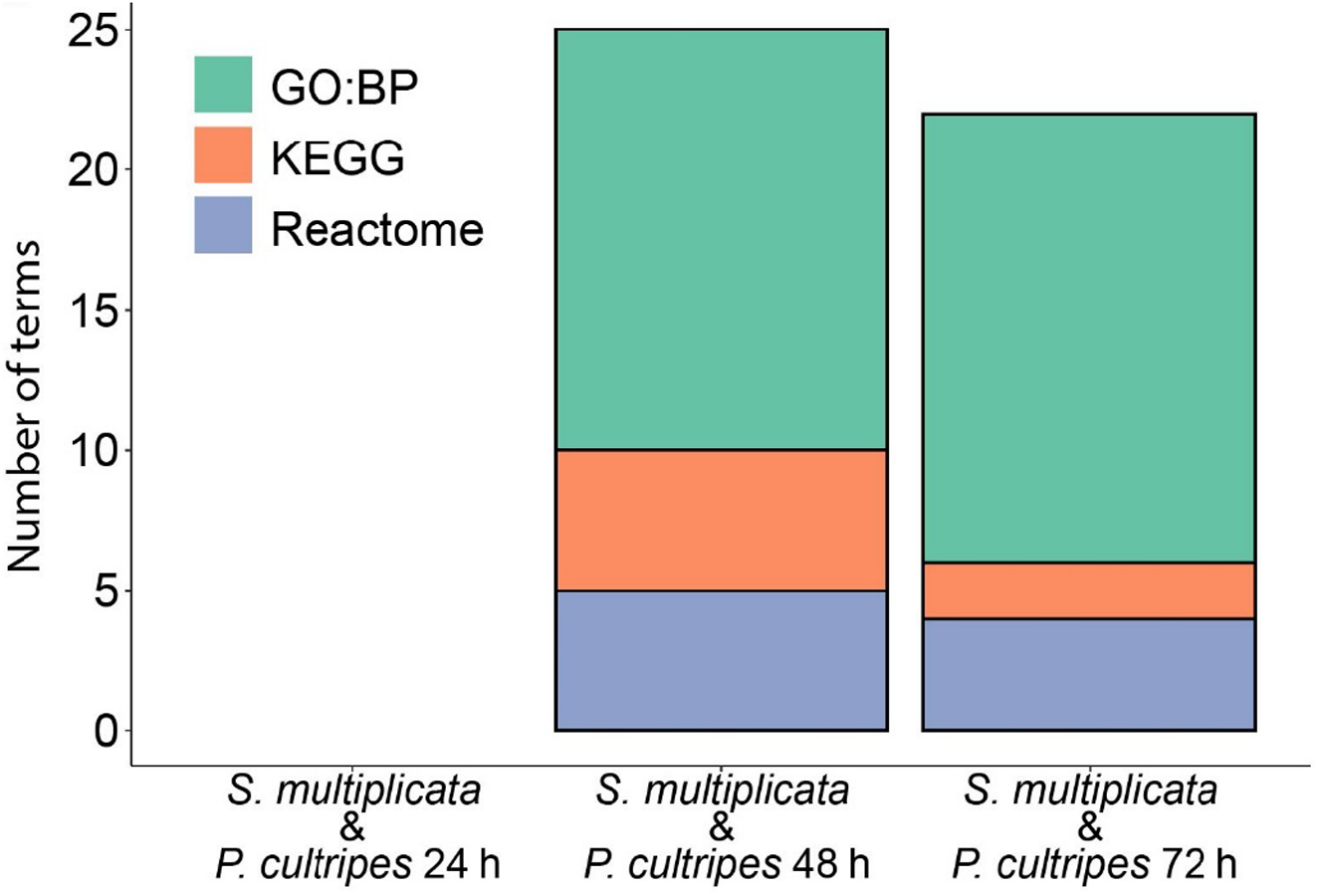
Overlapping functional annotation between *Spea multiplicata* and *Pelobates cultripes*. Stacked bar plot of overlapping functional annotation of the set of overlapping differentially expressed genes, including annotations from the GO:Biological Process, KEGG, and Reactome databases. Each bar is based on the list of overlapping genes between carnivores and omnivores in *S*. *multiplicata* and the genes from a comparison between the 24‐h high‐water control and the low‐water treatment stated along the *x*‐axis (24‐, 48‐, and 72‐h low‐water treatments, from left to right).

When we queried whether there were shared processes among the overlapping genes between carnivores and omnivores and for each time period (i.e., the set of shared DEGs between species comparisons), we found that these genes were enriched for particular functional terms at the latter two time points (Figure 5; Table 5). Specifically, when comparing *S*. *multiplicata* with *P*. *cultripes* using the 48‐h low‐water treatment, shared processes based on the shared DEGs include terms related to steroid metabolic processes, carbon metabolic processes, pyruvate metabolic processes, steroid biosynthesis, cholesterol biosynthesis, and tRNA aminoacylation. Comparing *S*. *multiplicata* with *P*. *cultripes* using the 72‐h low‐water treatment yielded some of the same (and similar) functionally enriched terms, including steroid metabolic processes, steroid biosynthesis, and cholesterol biosynthesis. Additionally, terms for cholesterol metabolic processes, lipid biosynthesis, and lipid metabolism were enriched at this time point. Thus, although the number of overlapping DEGs is not greater than expected, those that overlap are functionally enriched for putatively important biological processes.

**TABLE 5:** Summary of GO:Biological Process (GO:BP), KEGG, and Reactome (REAC) terms identified through functional annotation analysis of the overlapping genes between carnivores and omnivores in *Spea multiplicata* and high and low water at each time‐point treatment in *Pelobates cultripes*.

*P*. *cultripes* treatment	Source	GO term	Term name	Corrected *p*‐value
48 h low water	GO:BP	GO:0044281	Small molecule metabolic process	9.56E‐10
	GO:BP	GO:0019752	Carboxylic acid metabolic process	1.60E‐07
	GO:BP	GO:0043436	Oxoacid metabolic process	2.31E‐07
	GO:BP	GO:0006082	Organic acid metabolic process	2.55E‐07
	GO:BP	GO:0006520	Cellular amino acid metabolic process	4.18E‐05
	GO:BP	GO:0044283	Small molecule biosynthetic process	4.41E‐04
	GO:BP	GO:1901566	Organonitrogen compound biosynthetic process	6.79E‐04
	GO:BP	GO:0006418	tRNA aminoacylation for protein translation	1.39E‐03
	GO:BP	GO:0006399	tRNA metabolic process	1.66E‐03
	GO:BP	GO:0043039	tRNA aminoacylation	1.99E‐03
	GO:BP	GO:0043038	Amino acid activation	2.17E‐03
	GO:BP	GO:1902653	Secondary alcohol biosynthetic process	3.25E‐03
	GO:BP	GO:0006695	Cholesterol biosynthetic process	3.25E‐03
	GO:BP	GO:1901617	Organic hydroxy compound biosynthetic process	4.22E‐03
	GO:BP	GO:0016126	Sterol biosynthetic process	5.37E‐03
	KEGG	KEGG:00100	Steroid biosynthesis	1.02E‐05
	KEGG	KEGG:01100	Metabolic pathways	1.59E‐05
	KEGG	KEGG:00970	Aminoacyl‐tRNA biosynthesis	2.75E‐04
	KEGG	KEGG:01200	Carbon metabolism	5.85E‐04
	KEGG	KEGG:00620	Pyruvate metabolism	1.46E‐02
	REAC	REAC:R‐HSA‐191273	Cholesterol biosynthesis	1.01E‐06
	REAC	REAC:R‐HSA‐379716	Cytosolic tRNA aminoacylation	1.15E‐04
	REAC	REAC:R‐HSA‐8957322	Metabolism of steroids	5.78E‐04
	REAC	REAC:R‐HSA‐379724	tRNA Aminoacylation	1.17E‐03
	REAC	REAC:R‐HSA‐2426168	Activation of gene expression by SREBF (SREBP)	4.53E‐02
72 h low water	GO:BP	GO:0006695	Cholesterol biosynthetic process	3.38E‐05
	GO:BP	GO:1902653	Secondary alcohol biosynthetic process	3.38E‐05
	GO:BP	GO:0016126	Sterol biosynthetic process	5.61E‐05
	GO:BP	GO:0006694	Steroid biosynthetic process	5.78E‐05
	GO:BP	GO:0008610	Lipid biosynthetic process	1.03E‐04
	GO:BP	GO:1901617	Organic hydroxy compound biosynthetic process	2.72E‐04
	GO:BP	GO:1901615	Organic hydroxy compound metabolic process	6.27E‐04
	GO:BP	GO:0044283	Small molecule biosynthetic process	6.82E‐04
	GO:BP	GO:0008202	Steroid metabolic process	1.15E‐03
	GO:BP	GO:0008203	Cholesterol metabolic process	1.53E‐03
	GO:BP	GO:0046165	Alcohol biosynthetic process	1.57E‐03
	GO:BP	GO:1902652	Secondary alcohol metabolic process	2.03E‐03
	GO:BP	GO:0006066	Alcohol metabolic process	2.22E‐03
	GO:BP	GO:0016125	Sterol metabolic process	2.38E‐03
	GO:BP	GO:0006629	Lipid metabolic process	9.48E‐03
	GO:BP	GO:1900222	Negative regulation of amyloid‐beta clearance	1.70E‐02
	KEGG	KEGG:01100	Metabolic pathways	1.70E‐05
	KEGG	KEGG:00100	Steroid biosynthesis	5.74E‐05
	REAC	REAC:R‐HSA‐191273	Cholesterol biosynthesis	2.37E‐04
	REAC	REAC:R‐HSA‐8957322	Metabolism of steroids	1.30E‐03
	REAC	REAC:R‐HSA‐211945	Phase I—Functionalization of compounds	2.01E‐02
	REAC	REAC:R‐HSA‐556833	Metabolism of lipids	4.36E‐02

*Note*: The treatment in *P*. *cultripes* used for each set of annotations is in the first column (note that there are no significant terms when using the 24‐h low‐water treatment in *P*. *cultripes*).

### 3.4 Lineage‐specific gene expression plasticity in *S*. *multiplicata*

When comparing the DEGs in *S*. *multiplicata* to the genes that are not significantly differentially expressed in *P*. *cultripes*, we found that 2860 genes overlapped for the 24‐h low‐water treatment comparison, 2829 genes overlapped for the 48‐h low‐water treatment comparison, and 2855 genes overlapped for the 72‐h low‐water treatment (Figure 3). Additionally, a number of DEGs in *S*. *multiplicata* do not align to any gene in *P*. *cultripes* after reciprocal‐best‐match annotation. These number approximately 1620 at each of the three time points (Figure 3). Together, this suggests that a large number of genes insensitive to pond drying/developmental acceleration in *Pelobates* are condition dependent in the context of *Spea*'s resource polyphenism.

Functional enrichment analysis of the set of DEGs in *S*. *multiplicata* overlapping with genes not significantly differentially expressed in *P*. *cultripes* returned many high‐level functional terms (Data S3), including terms for organismal, head, brain, and nervous system development; protein metabolism; and response to endogenous stimuli, commensurate with the large‐scale changes involved in the resource polyphenism. Likewise, the genes showing plasticity in *S*. *multiplicata* but that did not align to genes in *P*. *cultripes* were enriched for diverse terms, including brain, head, and nervous system development (Data S3). Together, this suggests that major developmental reorganization is involved in the resource polyphenism, but genes underlying these changes were either not plastic or did not align to transcripts in the ancestral pond drying response of *P*. *cultripes*. Generally, these findings do not support the hypothesis of co‐option, but suggest that lineage‐specific changes to gene expression dominate the *Spea* plastic response.

## 4 DISCUSSION

Using a de novo transcriptome for *S*. *multiplicata* and a previously published transcriptome and data for *P*. *cultripes*, we investigated the origins of gene expression plasticity associated with a novel larval resource polyphenism in *S*. *multiplicata* (Figure 1a,c). We found that this derived form of plasticity appears to have evolved primarily via lineage‐specific changes in gene expression as opposed to co‐opting mechanisms from an ancestral form of plasticity—accelerating larval development rate in response to pond drying (Figure 1b,c).

Specifically, we found that these two forms of plasticity share a minimal set of differentially expressed genes and that most genes showing morph‐biased expression in *S*. *multiplicata* were not associated with the pond drying response in *P*. *cultripes* (Figures 3 and 4). On the one hand, this finding was unexpected: the polyphenism in *S*. *multiplicata* is characterized by the developmentally accelerated carnivore morph (de la Serna Buzon et al., 2020; Pfennig, 1992a, 1992b). On the other hand, this polyphenism involves much more than just developmental acceleration. Indeed, we found that the set of genes showing plasticity in *S*. *multiplicata*, but not showing it in *P*. *cultripes*, is enriched for major organismal, head, and brain development terms. These data are therefore consistent with previous studies, which have shown that carnivores differ from omnivores behaviorally (Pfennig, 1999; Pfennig et al., 1993; Pomeroy, 1981), morphologically (Levis et al., 2018; Martin & Pfennig, 2009; Pfennig, 1992b; Pfennig & Murphy, 2000, 2002), and physiologically (Ledón‐Rettig, 2021; Ledón‐Rettig et al., 2008, 2009, 2023). Thus, it makes sense that extensive lineage‐specific gene expression plasticity has evolved in *Spea*'s polyphenism when compared to the relatively simple plasticity of developmental acceleration in *Pelobates*.

Given our finding of few shared responses and extensive lineage‐specific responses, we speculate that the evolution of this polyphenism may have expanded from a limited set of shared plastic responses that are functionally enriched for having roles in lipid metabolism (especially cholesterol biosynthesis), steroid biosynthesis, and tRNA aminoacylation (Figure 5; Table 5). Subsequently, previously non‐plastic genes may have been recruited as the polyphenism underwent elaboration and refinement (Casasa et al., 2020; Foquet et al., 2021; Morris et al., 2014). Such a process may be especially likely to occur when, as suggested elsewhere (Levis et al., 2021, 2022), the shared responses constitute a core set of genes that promote a tadpole's development into alternative trajectories, and when the lineage‐specific plasticity genes constitute those that maintain, elaborate, and refine the alternative phenotypes (Lafuente & Beldade, 2019). Indeed, the evolution of polyphenisms in other taxa involves bringing other developmental processes into a conditionally expressed context (Abouheif & Wray, 2002; Bhardwaj et al., 2020; Hanna & Abouheif, 2022; Projecto‐Garcia et al., 2017; Sommer, 2020; Suzuki & Nijhout, 2006). Thus, we speculate that plasticity in a small set of genes and processes might set a lineage on the path to evolving a polyphenism, but substantial lineage‐specific alterations are needed for a polyphenism to actually arise.

Our results come with caveats. First, using whole tadpoles might obscure additional responses at individual tissue levels. We used whole tadpoles to ensure that our de novo transcriptome and analyses were similar to those of the previous study we were using as a reference (Liedtke et al., 2021). Additionally, the polyphenism in *S*. *multiplicata* involves a mosaic of tissues throughout the body, including the gut, jaw muscles, and brain (see above). Yet, future work would benefit from taking a tissue‐specific look at the development of both forms of plasticity, especially given the evidence from this system (Levis et al., 2022) and other systems (Mateus et al., 2014; Oostra et al., 2018; Suzuki & Nijhout, 2006; van der Burg & Reed, 2021) that tissues differ in how they respond to internal and external environmental change. Another caveat concerns the limited temporal sampling. If the omnivore‐to‐carnivore transition was assayed sooner (or later), or the response to pond drying was assayed sooner (or later), there may have been more similarities between the two forms of plasticity. As we have no a priori reason to believe any particular timepoint in the *P*. *cultripes* data is more similar to the *S*. *multiplicata* data, we compared all timepoints here, but future studies would benefit from more precise matching of the timeframe of development. Finally, given that *Spea* (like *Pelobates*) exhibits pond‐drying plasticity (Figure 1b,c), future studies should replicate the *Pelobates* experiment in *Spea* and determine the patterns their developmental rate plasticity generated. In doing so, one could identify which differentially expressed genes are related to developmental speed per se and not *Pelobates*‐specific plasticity. Thus, future studies could benefit from fine‐grained tissue, temporal, and lineage‐specific sampling to characterize further the degree to which these two forms of plasticity share transcriptomic bases.

With such future analyses in mind, the transcriptome assembled here provides a significant resource for *Spea*. For example, it will facilitate analyses of splicing and regulatory differences between morphs, investigating expression differences related to sexual selection and hybridization (Chen & Pfennig, 2020; Pfennig, 2007; Seidl et al., 2019), and the transcriptional bases of other aspects of *Spea* biology (Levis et al., 2021, 2022). Additionally, as demonstrated here, the transcriptome will allow for comparative studies of plasticity not only across other spadefoot species, but also more widely among Anura and higher taxa. This transcriptome provides a significant addition to the growing genomic resources available for *S*. *multiplicata*, which to date had no full‐length transcriptome‐wide annotation to accompany its assembled genome (Seidl et al., 2019). Moreover, it helps fulfill calls for more such resources in anurans generally (Kosch et al., 2023).

In conclusion, our results provide important insights into a novel polyphenism's evolutionary and developmental origins. The number of genes shared between an ancestral plastic response to pond drying via developmental acceleration in *P*. *cultripes* and the more complex polyphenism in *Spea* is dwarfed by the much greater number of genes gaining plasticity in *Spea*. These lineage‐specific gene expression patterns are involved in major developmental shifts that support the complex whole organism changes involved in carnivore production. Consistent with gene expression plasticity evolution in other systems (Casasa et al., 2020; Foquet et al., 2021), we also found that *Spea*'s polyphenism requires more gene expression changes than the pond drying response in *Pelobates*. Together, this suggests that more general ancestral stress responses might be a springboard for subsequent evolutionary innovation, but that substantial lineage‐specific modification is needed to craft such responses into an adaptive polyphenism. More generally, our work suggests that the evolution of complex forms of plasticity (like resource polyphenism) may have little reliance on simpler forms of ancestral plasticity, which could explain why polyphenisms are relatively rare across the tree of life.

## AUTHOR CONTRIBUTIONS

**Andrew J. Isdaner:** Data curation (lead); formal analysis (lead); investigation (lead); methodology (equal); resources (equal); validation (lead); visualization (equal); writing – original draft (equal); writing – review and editing (equal). **Nicholas A. Levis:** Conceptualization (lead); formal analysis (supporting); investigation (supporting); methodology (equal); project administration (supporting); supervision (supporting); visualization (supporting); writing – original draft (equal); writing – review and editing (equal). **David W. Pfennig:** Formal analysis (supporting); investigation (supporting); methodology (equal); project administration (lead); resources (equal); supervision (lead); validation (supporting); visualization (equal); writing – original draft (equal); writing – review and editing (equal).

## Supporting information


Data S1–S3.

## Data Availability

All raw sequences reads are available in the NCBI SRA (SRP339994). The transcriptome assembly has been deposited at DDBJ/EMBL/GenBank under the accession GKIA00000000. Both the raw sequence reads and the transcriptome assembly can be found in BioProject PRJNA768487. Data S1–S3 may be found published alongside this paper.
