# Supplementary material for: Comparative transcriptomics reveals that a novel form of phenotypic plasticity evolved via lineage‐specific changes in gene expression
Source: Ecol Evol. 2023 Oct 20;13(10):e10646. doi: 10.1002/ece3.10646 (PMC10589077; doi:10.1002/ece3.10646)
Supplement: Supplementary file 1 — Data S1–S3. [file ECE3-13-e10646-s001.zip › ece310646-sup-0004-Legends.docx]

**Supplemental Data 1**

Trinotate annotation of the S. multiplicata transcriptome assembly

**Supplemental Data 2**

Differentially expressed genes between: 1) carnivores and omnivores in S. multiplicata, 2) 24-hour high water control tadpoles and 24-hour low water treatment tadpoles in P. cultripes, 3) 24-hour high water control tadpoles and 48-hour low water treatment tadpoles in P. cultripes, and 4) 24-hour high water control tadpoles and 72-hour low water treatment tadpoles in P. cultripes.

**Supplemental Data 3**

Functional annotation terms for genes differentially expressed between morphs in S. multiplicata that were not significantly differentially expressed in P. cultripes for each of the comparisons between the 24-hour high water control and: 1) the 24-hour low water treatment, 2) the 48-hour low water treatment, and 3) the 72-hour low water treatment. There are, additionally, functional annotation terms for those genes that were differentially expressed between morphs in S. multiplicata that do not align to genes in P. cultripes, including that set of genes relative to the genes included in each of the three timepoint comparisons above [tabs 4), 5), and 6), respectively]. All functional terms were based on human gene annotations against the human background set.
